# Baicalein Inhibits α-Melanocyte-stimulating Hormone-stimulated Melanogenesis via p38 Mitogen-activated Protein Kinase Pathway in B16F10 Mouse Melanoma Cells

**DOI:** 10.15430/JCP.2023.28.2.40

**Published:** 2023-06-30

**Authors:** Min Chang Oh, Pincha Devage Sameera Madushan Fernando, Mei Jing Piao, Kyoung Ah Kang, Herath Mudiyanselage Udari lakmini Herath, Jin Won Hyun

**Affiliations:** 1 Department of Biochemistry, Jeju National University College of Medicine, Jeju, Korea; 2 Jeju Research Center for Natural Medicine, Jeju National University, Jeju, Korea

**Keywords:** α-Melanocyte-stimulating hormone, Baicalein, Carcinogenesis, Monophenol monooxygenase, B16F10 cells

## Abstract

Excessive UVB exposure causes development of both malignant and non-malignant melanoma via the secretion of α-melanocyte-stimulating hormone (α-MSH). We investigated whether baicalein (5,6,7-trihydroxyflavone) could inhibit α-MSH-stimulated melanogenesis. Baicalein prevented UVB- and α-MSH-induced melanin production and attenuated α-MSH-stimulated tyrosinase (monophenol monooxygenase) activity, and expression of tyrosinase and tyrosine-related protein-2. In addition, baicalein prevented melanogenesis and pigmentation via the p38 mitogen-activated protein kinases signaling pathway. These findings suggest that baicalein represents a natural compound for attenuating melanogenesis.

## INTRODUCTION

Melanin is produced from the epidermal melanocytes through a process called melanogenesis [1]. Melanogenesis of the skin provides a lot of valuable cytoprotective functions as melanin is needed for shielding the skin against the damaging effects of sunlight and chemical toxicants [2]. UV radiation exposure is considered to be the main cause of melanogenesis [3]. Furthermore, melanin is considered as the main determinant of hair, skin and eye colour, and has a vital function in protection against UV radiation [4]. However, it has been reported that UV radiation causes the overproduction of melanin, which leads to hyperpigmentation and other skin disorders such as ephelides (freckles), melasma, solar lentigines, pigmented acne scars, and cancer [5-7]. Notably, the dangerous form of skin cancer known as melanoma is characterized by an abnormally elevated level of melanin synthesis by melanocytes, and chronic exposure to excessive UV light leads to the development of both malignant and non-malignant melanoma [8].

It has been reported that UV radiation induces the secretion of α-melanocyte-stimulating hormone (α-MSH) which binds to the melanocortin 1 receptor (MC1R) [9,10]. Moreover, UV exposure induced melanogenesis by activating enzymes including, tyrosinase (monophenol monooxygenase), tyrosine-related protein-1 (TRP-1), TRP-2, and protein kinase C [11-14]. In addition, UV irradiation increases melanogenesis through direct effects on melanocytes as well as indirect effects on keratinocytes that release melanogenic components [15]. Furthermore, as a response to UV radiation-mediated DNA damage in keratinocytes, α-MSH is secreted in a p53-dependent manner, which binds to MC1R in melanocytes to produce melanin [16]. In detail, the α-MSH activates the MC1R, which activates the cyclic adenosine monophosphate signaling pathway, mainly associated with differentiation and pigment formation [17].

In general, melanogenesis increases the expression of microphthalmia-associated transcription factor (MITF) by activating the tyrosinase enzyme, TRP-1 or TRP-2, as well as tyrosinase activity, which results in enhanced melanin synthesis [18]. Tyrosinase, which contains copper, is the rate-limiting enzyme in the production of melanin [19]. The enzyme is responsible for catalyzing the biosynthesis of melanin whereas the hydroxylation of L-tyrosine to L-3,4-dihydroxyphenylalanine (DOPA) and then to DOPA-quinone. DOPA-quinone undergoes a redox exchange, being convereted into DOPA-chrome [20,21]. TRP-2 transforms DOPA-chrome to 5,6-dihydroxyindole-2-carboxylic acid (DHICA). TRP-1 oxidizes DHICA to a carboxylated indole-quinone, which then gets transformed to melanin [22]. The mitogen-activated protein kinase (MAPK) pathway connects cell-surface receptors to transcription machinery, converting extracellular signals into a variety of outcomes [23]. It has been reported that the p38 MAPK pathway regulates the melanogenesis; whereas the phosphorylation of p38 MAPK promotes melanin production by elevating MITF and tyrosinase expression [24].

Baicalein (5,6,7-Trihydroxyflavone), a flavone compound, is originally isolated from the roots of *Scutellaria baicalensis*. We have previously reported that baicalein protects cellular components against oxidative damage by scavenging reactive oxygen species (ROS), inhibiting apoptosis, and attenuating oxidative stress-induced expression of matrix metalloproteinase-1 in human keratinocytes [25,26]. Furthermore, our previous studies have demonstrated that baicalein has the ability to inhibit mitochondrial oxidative stress by activating transcription factor NF-E2-related factor 2 (Nrf2)-mediated induction of manganese superoxide dismutase expression [27]. Also, we have found that baicalein has a protective effect against UVB radiation-induced damage and apoptosis of human HaCaT keratinocytes [28]. In this study, we evaluated the inhibitory effects of baicalein on melanogenesis and investigated the mechanism underlying its anti-melanogenic activity in B16F10 cells.

## MATERIALS AND METHODS

### Reagents

Baicalein, [3-(4,5-dimethylthiazol-2-yl)-2,5-diphenyltetrazolium] bromide (MTT), α-MSH, L-DOPA, anti-actin antibody, arbutin and SB203580 (p38 inhibitor) were provided from Sigma-Aldrich Co. Antibodies against tyrosinase, TRP-2, and p38 were obtained from Santa Cruz Biotechnology. Antibody targeting phospho-p38 was purchased from Cell Signaling Technologies. All remaining chemicals and reagents were of analytical grade.

### Cell culture

B16F10 cells were provided by the American Type Culture Collection, and cells were maintained at 37°C in a humidified atmosphere of 5% CO_2_. The cells were cultured in Dulbecco’s modified eagle medium supplemented with 10% FBS, 100 µg/mL streptomycin, and 100 units/mL penicillin.

### Cell viability

Cells were cultured at a density of 0.5 × 10^5^ cells/well in a 24-well plate. Baicalein (1, 5, 10, and 20 μM) was added to the cells after 16 h of incubation at 37°C. Thereafter, 100 µL of a 2 mg/mL MTT stock solution was added to each well to generate a total reaction volume of 500 µL, and the cells were incubated for 4 hours. The plate was then centrifuged at 800 × *g* for 5 minutes, and the supernatants were removed. Formazan crystals were dissolved in dimethylsulfoxide (DMSO) and the absorbance at 540 nm was read using a scanning multi-well spectrophotometer [29].

### UVB-induced melanin content assay

Following a 1 hour treatment with 20 µM baicalein, cells were exposed to 30 mJ/cm^2^ of UVB radiation. Our previous study revealed that UVB exposure at 30 mJ/cm^2^ dose dependently increased the melanin content in B16F10 cells [30]. After 24 hours, the cells were detached by incubation in the presence of trypsin/ethylene diamine tetra-acetic acid, and centrifuged at 13,000 rpm for 5 minutes. The resulting cell pellets were solubilized at 80°C for 60 minutes in 1 N NaOH/10% DMSO solution. Melanin levels were determined by spectrophotometric analysis at 405 nm absorbance. The calculated index of melanin content represents as the index of optical density.

### α-MSH-induced melanin content assay

Cells were pre-treated for an additional 12 hours with 1 µM α-MSH. Cells were then treated with baicalein at a concentration of 20 µM or SB203580 at 1 µM. Following 24 hours of incubation, the cells were dissociated in trypsin/ethylene diamine tetra-acetic acid, centrifuged at 5,000 × *g* for 5 minutes, and then solubilized in 1 N NaOH/10% DMSO at 80°C for 60 minutes. Melanin levels have been determined by spectrophotometric analysis at an absorbance of 405 nm. The calculated index of melanin content represents as the index of optical density.

### Measurement of tyrosinase activity

Cells were pre-treated with 1 µM α-MSH for 12 hours. Thereafter, baicalein (20 µM), SB203580 (1 µM) or arbutin (1 mM) was added to the cells. Following 24 hours of incubation, the cells were lysed with 10% Triton X-100 in 80 mM phosphate buffer (pH 6.8) and then subjected to centrifugation at 12,000 rpm for 15 minutes at 4°C to isolate tyrosinase substrate solution as a supernatant. Then, 10 mM L-DOPA in phosphate buffer was mixed with the tyrosinase substrate solution and incubated at 37°C for 30 minutes. Tyrosinase activity was determined by spectrophotometric measurement of the absorbance at 570 nm.

### Western blotting analysis

Cells were seeded at 1.0 × 10^5^ cells/mL, and then, pre-treated with 1 µM α-MSH for 12 hours. Thereafter, baicalein (20 µM) or SB203580 (1 µM) was added to the cells. After incubation for 24 hours, cells were collected and washed twice with PBS. The protein concentrations were measured in the supernatants recovered from the lysates. The lysates were boiled for 5 minutes before being electrophoresed on 10% sodium dodecyl sulfate polyacrylamide gels. Blots were transferred onto nitrocellulose membranes (Bio-Rad) for blotting, which was subsequently treated with primary antibodies (tyrosinase, TRP-2, phospho-p38, p38, and actin). Secondary immunoglobulin G-horseradish peroxidase conjugates (Pierce) were added to the membranes, and then they were subjected to exposure X-ray film. Furthermore, using an improved chemiluminescence western blotting detection kit (Amersham), the corresponding protein bands were observed [31].

### Statistical analysis

All measurements were conducted in triplicate, and all values are expressed as the mean ± standard error. The results were analyzed using an analysis of variance (ANOVA) and Tukey’s test to determine the differences between means. In each instance, a *P*-value of 0.05 was regarded as statistically significant. Statistical analysis was performed with SigmaPlot 12 (Systat software).

## RESULTS

### Baicalein attenuates UVB- and α-MSH-induced melanin synthesis

**Figure 1:**
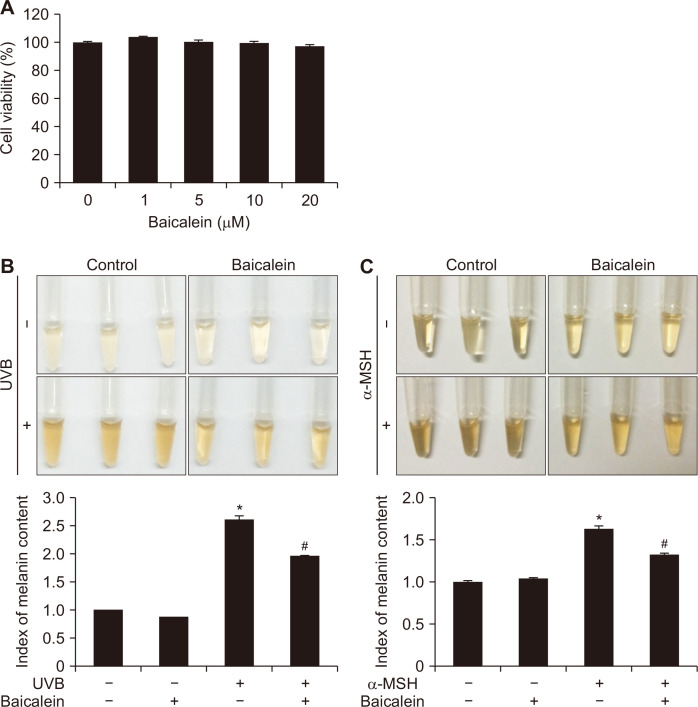
Baicalein inhibits UVB- and α-MSH induced enhancement of melanin contents in B16F10 cells. (A) The cytotoxic effect of baicalein at concentrations of 0, 1, 5, 10, or 20 µM was determined by the MTT assay. (B) UVB radiation was used to determine the amount of melanin. Cells were pretreated with baicalein for 1 hour, then UVB was irradiated. Spectrophotometric analysis at 405 nm absorbance determined the melanin concentration. *P < 0.05 and ^#^*P* < 0.05 compared to the control and UVB-exposed cells, respectively. (C) After pre-treatment of cells with 1 µM α-MSH for 12 hours, baicalein was applied to the cells. The melanin content was analyzed spectrophotometrically by measung absorbance at 405 nm. α-MSH, α-melanocyte-stimulating hormone; MTT, [3-(4,5-dimethylthiazol-2-yl)-2,5-diphenyltetrazolium] bromide. *P < 0.05 and ^#^*P* < 0.05 compared to the control and α-MSH-treated cells.

First, baicalein cytotoxicity to B16F10 cells was assessed by the MTT assay. Baicalein at concentrations of 1, 5, 10, and 20 µM did not cause any significant cytotoxicity during incubation for 24 hours (Fig. 1A). Thus, in all following experiments, baicalein was used at 20 µM. To investigate the role of baicalein in melanogenesis, we examined UVB- and α-MSH-induced melanin synthesis. As shown in Figure 1B, the melanin contents of the UVB-treated group significantly increased as evidenced by the higher index of melanin content (2.6) compared to the non-treated control group (the index of melanin content; 1.0). However, the index of the melanin content (2.0) of the baicalein pre-treated and UVB-exposed group was significantly reduced compared to the UVB alone exposed group. Further, the index of the melanin content of the α-MSH pre-treated group (1.6) was significantly increased compared to the non-treated control group (the index of melanin content; 1.0). The index of the melanin content of α-MSH pre-treated and baicalein-treated groups (1.3) was significantly reduced to below the levels of the α-MSH pre-treated group (Fig. 1C). These results suggest that baicalein can reduce UVB- and α-MSH-stimulated melanogenesis in B16F10 cells.

### Baicalein inhibits α-MSH-stimulated tyrosinase activity and tyrosinase-related protein expression

**Figure 2:**
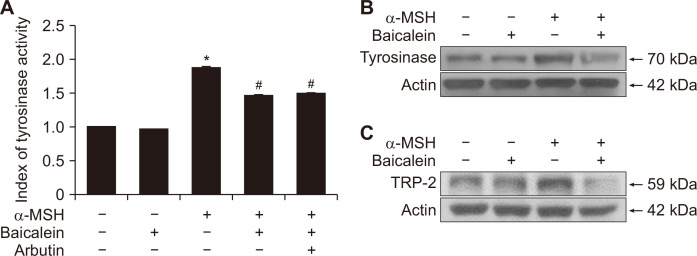
Baicalein attenuates α-MSH-stimulated tyrosinase activity and tyrosinase-related protein expression. (A) The 1 µM α-MSH was treated to the cells for 12 hours, then baicalein or arbutin was added. Spectrophotometric analysis measuring absorbance at 570 nm was conducted to determine tyrosinase activity. *P < 0.05 and ^#^*P* < 0.05 compared to the control and α-MSH-treated cells. The Western blot analysis was performed by using (B) tyrosinase antibody and (C) TRP-2 antibody, with actin used as the loading control. α-MSH, α-melanocyte-stimulating hormone; TRP, tyrosine-related protein.

We measured L-DOPA oxidation utilizing cell lysates as a source of tyrosinase to determine the effect of baicalein on cellular tyrosinase activity. The cells were pre-treated with 1 µM α-MSH and then treated with baicalein or arbutin as a representative tyrosinase inhibitor [32]. As shown in Figure 2A, the index of tyrosinase activity was significantly increased in the α-MSH pre-treated group (1.9) compared to the non-treated control group (index of tyrosinase activity; 1). However, the α-MSH-induced index of tyrosinase activity was significantly decreased in the baicalein or arbutin-treated group, with 1.4 of index of tyrosinase activity in the baicalein-treated group and 1.5 of index of tyrosinase activity in the arbutin-treated group (Fig. 2A).

Moreover, to investigate the expression of tyrosinase and tyrosinase-related protein, we performed Western blot analysis. As shown in Figure 2B, the protein levels of tyrosinase in the α-MSH-stimulated group were increased; however, the tyrosinase level induced by α-MSH-stimulated group was reduced by baicalein treatment. The protein levels of TRP-2 in the α-MSH-stimulated group were increased, which was reduced by baicalein treatment (Fig. 2C). These results suggest that baicalein reduces pigmentation in α-MSH-stimulated B16F10 cells by inhibiting the tyrosinase activity and expression of tyrosinase and TRP-2.

### Baicalein prevents melanogenesis and pigmentation via the p38 MAPK pathway

**Figure 3:**
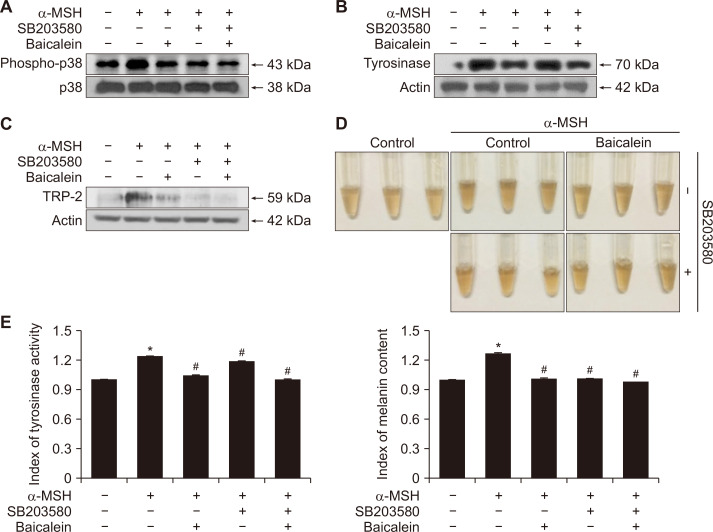
Baicalein prevents melanogenesis and pigmentation via the p38 MAPK pathway. The 1 µM α-MSH was treated to the cells for 12 hours, then baicalein or SB203580 was added. (A-C) The expression levels of the p38, phospho-p38, tyrosinase, and TRP-2 proteins were examined by Western blot analysis, with actin used as the loading control. (D) Melanin contents and (E) tyrosinase activity were measured with and without SB203580 and/or baicalein. *P < 0.05 and ^#^*P* < 0.05 compared to the control and α-MSH-treated cells. MAPK, mitogen-activated protein kinase; α-MSH, α-melanocyte-stimulating hormone; TRP, tyrosine-related protein.

We investigated the involvement of the p38 MAPK pathway in the melanogenesis and pigmentation. The protein level of phospho-p38 in the α-MSH-stimulated group was increased, and this increased phospho-p38 protein level was reduced by the p38 MAPK inhibitor SB203580 and baicalein (Fig. 3A). As shown in Figure 3B, the protein expression of tyrosinase in the α-MSH-stimulated group was increased compared to the non-treated control group, and this was attenuated by SB203580 and baicalein co-treatment. Moreover, the protein levels of TRP-2 in the α-MSH-stimulated group were increased compared to the non-treated control group, which was reduced by SB203580 and baicalein (Fig. 3C).

Lastly, we measured the melanin content and tyrosinase activity. The index of melanin content was significantly increased in the α-MSH-stimulated group (1.3) compared to the non-treated control group (1.0); however, α-MSH induced increase of melanin content was significantly reduced in the baicalein-treated group, the SB203580-treated group, and the SB203580 and baicalein co-treated group (Fig. 3D). The tyrosinase activity was increased in the α-MSH-stimulated group (1.3) compared to the untreated control group (1.0), which was significantly decreased by baicalein, SB203580, and SB203580 and baicalein in combination (Fig. 3E). Thus, baicalein directly or indirectly regulates melanogenesis and pigmentation by targeting the p38 MAPK activity.

## DISCUSSION

The α-MSH is a melanocortin peptide that is known to induce skin pigmentation [33]. α-MSH is a key cytokine that induces hyperpigmentation and is released by keratinocytes [34]. Furthermore, UVB exposure increases α-MSH release and induces hyperpigmentation due to excessive melanin synthesis [35]. In melanocytes, melanin is synthesized and stored in specialized organelles called melanosomes [36]. Melanosomes are generated via a sequence of well-defined steps. Throughout stages I and II of melanosome development, there is no pigmentation. In stage II melanosomes, internal striations are developed whereas fibrils are created completely. In the III stage, melanins are deposited on these fibrils, and during the stage IV, melanosomes are totally pigmented and matured [37,38]. Eventually, melanin-containing melanosomes are moved from the perinuclear region to the tips of dendrites in epidermal melanocytes, and then delivered to keratinocytes [39]. Pigmentary disorders, such as Hermansky–Pudlak syndrome, Chediak–Higashi syndrome, and Griscelli syndrome, are associated with melanogenesis and melanosome transport abnormalities [38]. Furthermore, excessive melanin production can result in hyperpigmentation and skin cancer [40].

Previous studies have shown that the p38 MAPK pathway can stimulate melanogenesis by elevating MITF expression, whereas, through binding to an M-box pattern in their promoter sites, MITF upregulates the production of tyrosinase, TRP-1, and TRP-2 [41,42]. Recently, many studies have reported that natural compounds have the potential to inhibit melanogenesis by downregulating the production of MITF and melanogenic enzymes through suppression of p38 MAPK [43,44]. Moreover, it has been reported that baicalein inhibits the melanogenesis through the ERK pathway activation and then further reduces melanin synthesis via MITF downregulation [45].

Our previous study showed that baicalein also has antioxidant potential [25]. Furthermore, in the present study, baicalein attenuated UVB and α-MSH-induced melanin production, tyrosinase activity, and expression of tyrosinase and TRP-2 as well as p38 MAPK phosphorylation, which is critical to MITF expression. In conclusion, baicalein represents a safe natural compound for inhibiting melanogenesis and a potential therapeutic candidate against UVB-induced excessive melanogenesis.
